# Thrombocytopenic purpura associated with common variable immunodeficiency and bronchiectasis

**DOI:** 10.1186/1939-4551-8-S1-A281

**Published:** 2015-04-08

**Authors:** Thais Martins Souza

**Affiliations:** 1 Ufjf, Brazil

## Introduction

Common variable immunodeficiency (CVID) is the most prevalent primary immunodeficiency (PID) and progresses with hypogammaglobulinaemia, recurrent infections and other prevalent alterations in humoral immunity. With respect to infections, pulmonary involvement stand and its complications

## Objective

Describe a case of patient with ICV who started with thrombocytopenia and evolved early with bronchiectasis.

## Case report

Boy with 13 years old diagnosed with thrombocytopenic purpura and a normal myelogram. He presented low levels of immunoglobulins (A = 7 mg / dl, M = 14,2mg / dl and G = 140 mg / dl). Immunophenotyping of lymphocytes was altered with CD19 = 7.9% (81cells / mm3); CD4 = 56.5% (574cells / mm3) and CD8 = 12.3% (125cells / mm3). The research of vaccine antibodies (tetanus, mumps) was negative and did not produce antibodies against the pneumococcal vaccine. In his personal history was report of pneumonia at age 12 and chicken pox infection with secondary prolonged evolution and secondary bacterial infection. A tomography of the chest showed signs of diffuse bronchiectasis. After excluding other causes of hypogammaglobulinemia and the diagnosis of CVID was initiated monthly treatment with intravenous immunoglobulin and physiotherapy.

## Conclusions

Reiterate the importance of diagnosis of bronchiectasis in patients with CVID, even under a single episode of pneumonia in evolution. These patients may have few signs and symptoms of respiratory and progress to complications, such as bronchiectasis, even in the presence of adequate monitoring. Furthermore, we emphasize the importance of investigating the causes of thrombocytopenic purpura associated with autoimmunity by some patients with CVID.

## Consent

Written informed consent was obtained from the patient for publication of this abstract and any accompanying images. A copy of the written consent is available for review by the Editor of this journal.

## References

[B1] Chapel H Cunningham-Rundles C Update in understanding common variable immunodeficiency disorders (CVIDs) and the management of patients with these conditions British journal of haematology 2009 145 6 709 27 10.1111/j.1365-2141.2009.07669.x 19344423 PMC2718064

[B2] Park MA Li JT Hagan JB Maddox DE Abraham RS Common variable immunodeficiency: a new look at an old disease Lancet 2008 372 9637 489 502 10.1016/S0140-6736(08)61199-X 18692715

